# The Impact of the COVID-19 Pandemic on Pediatric Dentistry: Insights from an Italian Cross-Sectional Survey

**DOI:** 10.3390/dj11060154

**Published:** 2023-06-19

**Authors:** Giulio Conti, Francesca Amadori, Alessandra Bordanzi, Alessandra Majorana, Elena Bardellini

**Affiliations:** 1 Department of Medicine e Surgery, School of Dentistry, University of Insubria—Italy Via Ravasi 2, 21100 Varese, Italy; giuliocontiphd@gmail.com; 2 Department of Medical and Surgical Sciences and Public Health, School of Pediatric Dentistry, University of Brescia, Italy Pl. Spedali Civili 1, 25123 Brescia, Italy; francesca.amadori@unibs.it (F.A.); bordanziale@gmail.com (A.B.); elena.bardellini@unibs.it (E.B.)

**Keywords:** paediatric dentistry, COVID-19, survey, childhood post graduated dental educational

## Abstract

Background. The COVID-19 pandemic has had a significant impact on pediatric dentistry and also dental education. This study aimed to assess the observed changes in children’s oral health by pediatric dentists and also served as an educational tool for dentistry students during the pandemic. Methods. Postgraduate students in pediatric dentistry prepared a survey addressed to Italian pediatric dentists. Over 5476 dentists were invited to participate, and collaboration among the students took place through virtual meetings and electronic platforms. The online questionnaire was composed of 29 questions focusing on the management of pediatric patients during and after the lockdown period. A descriptive statistic was used for data analysis, and chi-square tests were performed (*p* < 0.05). Results. A total of 1752 pediatric dentists participated in the survey. During the lockdown, 68.3% of dentists exclusively handled dental emergencies. In the subsequent semester, a significant decrease in the number of pediatric treatments was reported. Pediatric dentists also noted a decline in children’s oral hygiene practices, a deterioration in dietary habits, and an increase in anxiety during dental procedures. Conclusions. This survey shed light on the diverse effects of the pandemic on children’s oral health and also provided valuable educational insights.

## 1. Introduction

In 2020, Italy was the first Western nation seriously affected by the SARS-CoV-2 infection. Dentists have been identified as one the highest COVID-19 risk medical professions due to aerosol-generating procedures involved in dental treatment and the close proximity to patients’ mouths [1,2,3,4]. During the Italian lockdown from 9 March to 4 May 2020, many dental practitioners completely closed their practices and only provided urgent dental treatments due to the high risk of COVID-19 transmission for employees, dentists, and patients. Since 5 May, dental practices mostly resumed operations with additional triage measures and the use of personal protective equipment (PPE), such as FFP2 masks, gloves, gowns, goggles, or face shields. The COVID-19 outbreak has brought about significant changes in various aspects of dental practice. Government decisions and control measures have been implemented to limit the spread of the virus, including management protocols and telemedicine [5]. Several surveys [6,7,8,9] have been conducted during the pandemic period to understand dentist’s response to new challenges posed by COVID-19, but few of them have focused on pediatric dentistry. As a result of the reduced number of pediatric admissions to public facilities during the pandemic, the clinical training of students has been significantly reduced, leading to modifications and alternative teaching methods.

This survey, developed by students, aimed to investigate how Italian pediatric dentists handled the pandemic emergency during the lockdown and the post lockdown phase and to assess the observed changes in children’s oral health, as reported by pediatric dentists. Additionally, the survey served as a valuable teaching tool for the students involved.

## 2. Materials and Methods

### 2.1. Study Design

A cross-sectional study was designed to investigate the management of pediatric dental treatments during the Italian lockdown period (9 March 2020, to 4 May 2020) and after the end of the lockdown (5 May 2020).

### 2.2. Participants

Over 5476 dentists were invited to participate.

### 2.3. Survey Development

A class of 12 post graduate students in pediatric dentistry prepared the questionnaire. They discussed relevant topics, listed them, and formulated the survey questions for Italian pediatric dentists.

### 2.4. Questionnarie Validity

The questionnaire was pre-tested on eight randomly chosen pediatric dentists. Test–retest reliability was assessed using the Intraclass Correlation Coefficient (ICC), with a threshold of 0.80 considered satisfactory. Items with ICC below the threshold were modified.

### 2.5. Data Collection

An online self-administered questionnaire using Google Forms was distributed through mailing lists and social networks in December 2020. The survey consisted of 29 questions in the Italian language, covering demographic features, management during the lockdown period, and management during the post lockdown period.

### 2.6. Study Population

The study population included Italian pediatric dentists. A detailed analysis was conducted between two macro areas with different infection rates: regions with cumulative incidence > 40,000/100,000 (Lombardia, Piemonte, Trentino-Alto Adige, Valle d’Aosta, and Veneto) and regions with incidence < 4000/100,000 inhabitants (the other 15 regions).

### 2.7. Informed Consent

The questionnaire was anonymous and voluntary. Participants were required to provide online informed consent before proceeding with the questionnaire. Non-consenting participants were automatically excluded.

### 2.8. Data Analysis

Data were exported to an Excel file (Microsoft Corp., Redmond, WA, USA) and analyzed by STATA16 (Version 16.1 for Mac, Stata Corp., College Station, TX, USA). Descriptive statistics, including frequencies and percentages, were used to summarize the data. To compare the differences between the two macro-areas, chi-square tests were performed. Statistical significance was set at 5% (*p* < 0.05).

### 2.9. Ethics Approval

This study did not require ethics committee approval under Italian law.

## 3. Results

One thousand, seven hundred, and fifty-two pediatric dentists (1478 females and 274 males) participated in the survey. Approximately forty-nine per cent (*n* = 855) of respondents were aged between 31 and 49 years, 39.5% (*n* = 692) were 30 years or younger, 10.2% (*n* = 178) were aged between 50 and 60 years, and 1.5% (*n* = 27) were 61 years or older. Eight hundred and fifty-five dentists (48.8%) had a moderate level of professional experience. Around 55% (*n* = 964) of dentists were dental associated, 38.2% (*n* = 670) were dental owners, 3.5% (*n* = 61) were dental employees, and 3.3% (*n* = 57) were medical directors. The majority of the sample was located in North Italy (*n* = 1148, 65.5%), while the remaining 34.5% (*n* = 604) was distributed in the Center and the South of Italy, including the islands. On average, 54.7% (*n* = 958) of dentists treated more than 10 pediatric patients per week, while 24.5% (*n* = 430) managed between six and ten pediatric patients, and 20.8% (*n*= 364) treated less than six patients per week. None of the interviewees treated positive pediatric patients during the first Italian lockdown. After the first lockdown, only 3.5% (*n* = 61) of dentists treated positive or suspected pediatric patients. The characteristics of the respondents are presented in Table 1.

### 3.1. Management during the First Italian Lockdown (from 9 March to 4 May 2020)

From 9 March to 4 May 2020, 68.3% (*n* = 1196) of dentists managed dental emergencies. Significant differences were found between dentists located in different geographic areas, with varying levels of experience and with different working positions. In Northern Italy, 65.9% (*n* = 756) of dentists treated urgent pediatric dental care, while, in other regions, the percentage was 72.8% (*n* = 440) (*p* ≤ 0.0001). Dentists with high experience were more likely to handle urgencies compared to those with middle and low experience (*p* ≤ 0.0001). Dental associates and dental owners were more involved in treating pediatric patients compared to dental employees and medical directors (*p* ≤ 0.0001) (Table 2).

Around 82% (*n* = 980) of respondents handled one to five dental emergencies, while 15.2% (*n* = 182) managed between six and ten emergencies. In the North, 87.4% (*n* = 661) of dentists handled less than six emergencies, compared to 72.5% (*n* = 319) in other regions (*p* ≤ 0.0001). Dentists with high, middle, and low experience treated less than six emergencies, with 22.2% of dentists with middle experience handling between six and ten emergencies (*p* ≤ 0.0001). Dental associates, dental employees, and medical directors handled between one and five emergencies, while dental owners managed between six and ten emergencies (*p* ≤ 0.0001) (Table 2).

Regarding the request of dental alveolar trauma management, more than half of the dentists reported no change during the lockdown (Table 2). Among dentists located outside Northern Italy, 69.4% reported no change, while only 15.7% reported a decrease, and 14.9% (*n* = 90) reported an increase in dental alveolar trauma. In Northern Italy, 57.8% of dentists reported no change, while 34.0% observed a decrease in the number of dental alveolar treatments (*p* ≤ 0.0001). The decrease was more pronounced among dentists with high experience compared to those with low and middle experience (*p* ≤ 0.0001). Among dentists with moderate experience, 14% reported an increase in dental alveolar trauma.

Fourteen percent of dental owners observed an increase in dental alveolar trauma, while medical directors reported a decrease. Dental associates did not report any change in the number of dental alveolar traumas (*p* ≤ 0.0001).

### 3.2. Management of Paediatric Patients after the Lockdown

From May 2020 to January 2021, 21.5% dentists reported a decrease in pediatric patients. The decrease was more prevalent among dentists in Northern Italy, with 24.3% reporting a decrease compared to 16.2% (in other regions (*p* ≤ 0.0001). Among different working positions, 49.2% of dental employees, 23% of dental associates, and 18.7% of dental owners observed a reduction in pediatric patients. None of the medical directors reported a decrease in pediatric patients after the end of lockdown (Table 3).

During telephone triage (Table 3), 67% of dentists reported an increase in parents’ anxiety. The highest percentage of parents’ anxiety was reported in the North (76.5%, *n* = 878) (*p* ≤ 0.0001) among dentists with low level of experience and among dental associates and medical directors (*p* ≤ 0.0001) (Table 3).

Telephone triage and COVID-19 questionnaires were administered to both parents and pediatric patients without difference among geographic areas and, for parents, working position (Table 3). Approximately 92% of dentists with middle experience administered the COVID-19 questionnaire to both parents and pediatric patients, while 32.9% of dentists with low experience and 29.3% of dentists with high experience administered the COVID-19 questionnaire only to parents (*p* ≤ 0.0001).

During treatments, 56.6% (*n* = 991) of dentists allowed parents to stay in the dental treatment room only if requested. Parents stayed in waiting room for 30.6% of dentists in the other regions and 23.1% in the North. Only 18.9% of dentists in the North and 15.6% in the other regions allowed parents to stay in the dental treatment room as they did before the COVID-19 outbreak (*p* = 0.002). Forty-two per cent of dentists with high experience required parents to stay in the waiting room, while 73.3% and 49.6% of dentists with low and middle experience allowed parents to stay in the dental room treatments if requested (*p* ≤ 0.0001). More than half (*n* = 31) of dental employees required parents to stay in the waiting room during treatments, while 37.3% of dental owners allowed parents to stay in dental room treatments, as they did before the COVID-19 outbreak (*p* ≤ 0.0001) (Table 3).

Eighty-five percent of dentists always wore PPE during pediatric treatments. This safe approach was mainly applied by dentists in the other regions compared to dentists located in the North of Italy, by those with low and middle experience, and by all dental employees and medical directors (Table 3).

After the lockdown, 47% (*n* = 835) of dentists performed control visits, followed by 19.4% (who performed dental urgencies and 19% who performed first visits). Control visits were mainly performed by dentists in Northern Italy (50%, *n* = 574) compared to dentists in the other regions (43.2%, *n* = 261). About 25.5% of dentists in the other regions performed first visits. These differences were statistically significant (*p* ≤ 0.0001). Approximately 60% of dentists with low experience performed control visits, while only 39.5% and 42.9% of dentists with middle and high experience performed control visits. First visits and dental urgencies were predominantly performed in prevalence by 24.4% and 21.9% of dentists with middle experience (Table 3).

The use of rubber dams increased by 15.3% among dentists, remained unchanged for 81.1%, and decreased only for 3.7%.

**Figure 1:**
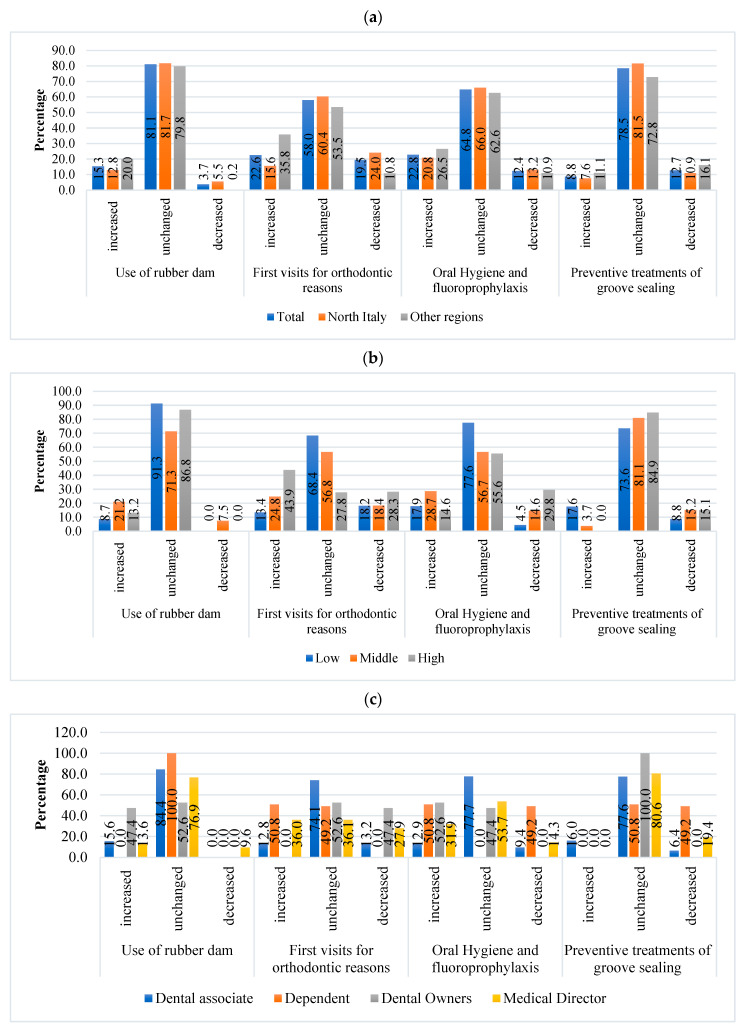
Dental treatments after the Italian lockdown—(**a**) comparisons among dentists located in Northern Italy or in the other regions; (**b**) comparisons among dentists with different experience levels; (**c**) comparisons among dentists with different roles in dental staffs.

Statistical differences emerged between geographic areas: the use of rubber dams increased among dentists in the other regions (20%) compared to those in the North (12.8%), where a higher reduction in rubber dam use was noted (5.5%) (*p* ≤ 0.0001)) (Figure 1a).

First visits for orthodontic reasons remained unchanged for 58% of dentists, mainly in Northern Italy (60.4%) compared to other regions (53.5%). A higher decrease in first visits for orthodontic reasons was observed in Northern Italy (Figure 1a).

Requests for oral hygiene and fluoroprophylaxis remained unchanged for 64.8% of dentists and increased especially among dentists located in the other regions compared to dentists in the North. A significant increase was observed among dentists with moderate experience (28.7%), while 29.8% of dentists with high experience reported a decrease in treatments for oral hygiene and fluoroprophylaxis (Figure 1b).

Preventive treatments of groove sealing remained unchanged for 78.5% (*n* = 1376) of dentists. This percentage was confirmed among dentists in the North (81.5%). In comparison, approximately 16% and 11.1% of dentists in the other regions reported a decrease and increase in these treatments (Figure 1a), respectively. Details about dental treatments, based on dentists’ experience or working position, are reported in Figure 1b,c.

**Figure 2:**
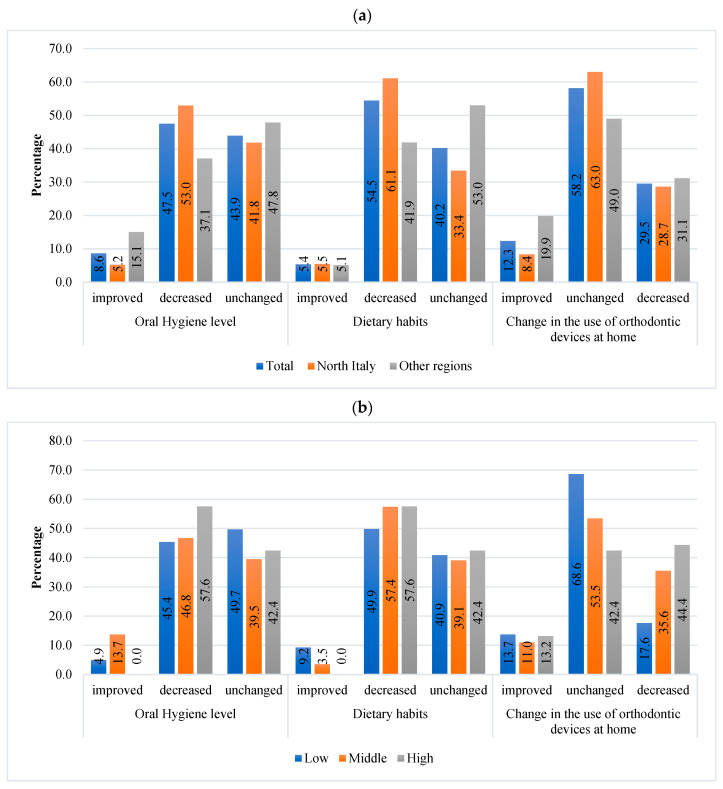
Oral hygiene and dietary habits during the COVID-19 pandemic—(**a**) comparisons among dentists located in Northen Italy or in the other regions; (**b**) comparisons among dentists with different levels of experience.

For 47.5% of dentists, the level of oral hygiene decreased during the COVID-19 pandemic. In the evaluation of oral hygiene in pediatric patients, statistically significant differences emerged among geographic areas. More dentists in Northern Italy (53%, *n* = 608) reported a decrease in oral hygiene, while 37.1% of those in the other regions reported such a reduction. For 15.1% of dentists in the other areas, the level of oral hygiene in pediatric patients increased during the pandemic (Figure 2a). Approximately 58% of dentists with high experience reported a decrease in oral hygiene, while about 13.7% of dentists with moderate experience noted an increase in the level of oral hygiene (Figure 2b).

For 55% (*n* = 954) dentists, good dietary habits decreased during the pandemic. This decrease was mainly observed by dentists in Northern Italy (61.1%) compared to those in the other regions (41.1%) (Figure 2a).

Dentists with high or moderate experience reported a decrease in dietary habits, while 9.2% of dentists with low experience reported an increase in dietary habits (*p* ≤ 0.0001) (Figure 2b).

Patients’ collaboration in using orthodontic devices at home remained unchanged for 58.2% (*n* = 1019) of dentists and decreased for 29.5% (*n* = 517) of the respondents. For 31.1% (of dentists in the other regions, the use of orthodontic devices at home decreased, while it increased for 19.9% during the pandemic (Figure 2a). This reduction in collaboration was mainly observed by 44.4% of dentists with high experience, while it was 68.6% of dentists with low experience and 53.5% for those with moderate experience, which reported no change (*p* ≤ 0.0001) (Figure 2b).

**Figure 3:**
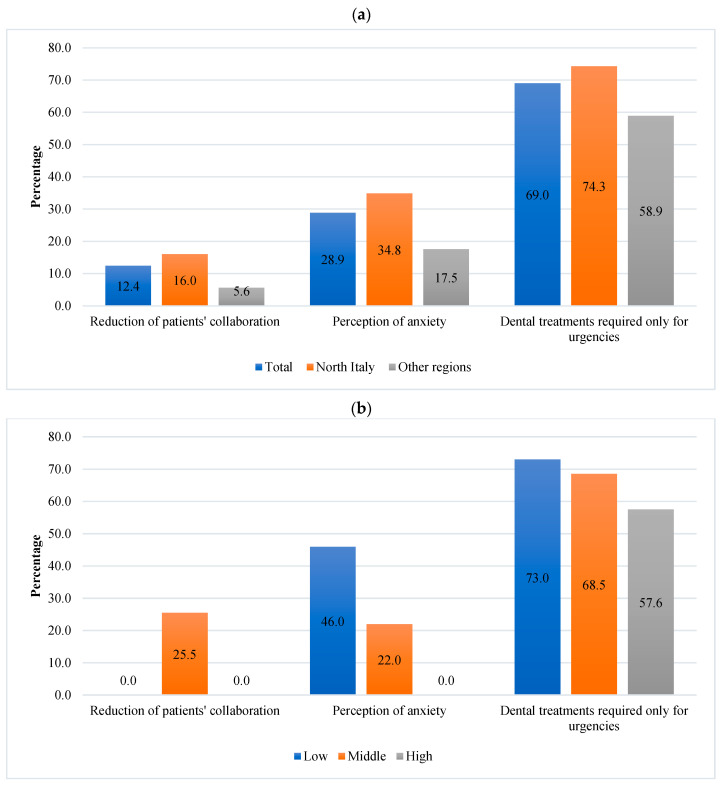
Patients’ collaboration and parents’ anxiety after the Italian lockdown—(**a**) comparisons among dentists located in Northen Italy or in the other regions; (**b**) comparisons among dentists with different levels of experience.

The reduction in patients’ collaboration was reported by only 12.4% of dentists, especially in Northern Italy compared to the other regions and among dentists with moderate experience (Figure 3b).

Approximately 28.9% (*n* = 506) of dentists perceived parents’ anxiety in accessing dental treatments. This perception was higher among dentists in Northern Italy (34.8%) compared to those in the other regions (17.5%) (Figure 3a), as well as among dentists with low experience (46%) compared to those with moderate (22%) or high (0%) experience (Figure 3b).

Sixty-nine (*n* = 1209) percent of dentists perceived that pediatric treatments were required only for emergencies. The perception was higher among dentists in Northern Italy compared to the other regions (Figure 3a), as well as among dentists with low experience (compared to those with moderate or high experience (*p* ≤ 0.0001) (Figure 3b).

## 4. Discussion

The COVID-19 pandemic has had profound effects on individuals and their interactions within society. As the outbreak initially unfolded, with high infection rates and significant mortality, many national authorities and dental organizations around the world implemented strict measures and restrictions on professional activities. Dental care, in particular, was limited to urgent cases in most countries during the early stages of the pandemic. The response of the dental profession to COVID-19 has varied across different regions. A global survey conducted by Campus et al. [10] assessed the impact of the pandemic on dental professionals in multiple countries worldwide. The findings revealed that, while routine dental care access was reduced due to temporary lockdown measures imposed by individual countries, the overall provision of oral health services was not significantly affected. This can be attributed, at least in part, to the preventive measures implemented by dental professionals globally to safeguard both themselves and patients from the risk of infection when treating symptomatic individuals. These measures have played a crucial role in mitigating the potential spread of the virus within dental settings and ensuring the safety of both dental practitioners and patients.

To the best of our knowledge, this is the first survey that represents the first attempt to provide an overview of Italian pediatric dentistry following the outbreak of COVID-19. Pediatric dentistry, a relatively recent field, focuses on educating children and about proper oral health practices, including issues related to dental caries, orthodontics, and anxiety and pain management [11].

This survey endorsed that pediatric dentistry is predominantly practiced by female and young dentists. The treatment of children, especially their first dental care experience, requires patience and empathy, which aligns with the characteristics often attributed to younger practitioners. The majority of the surveyed dentists were females under the age of 49, and especially those under 31 years old, who reported seeing more than ten pediatric patients each week.

During the lockdown, dental practices were completely closed, except for emergency cases. However, when regular activity resumed, noticeable differences were observed in management, both in terms dentists’ practices and patients’ attitudes.

A significant decrease in the number of pediatric patients was observed compared to the period before the spread of SARS-CoV-2, especially in the North of Italy. This decrease can be attributed to increased concern and anxiety surrounding dental care [12,13,14], as well as the economic impacts and uncertainties caused by the pandemic [15]. Financial concerns led many parents, especially those from the lowest-income households, to postpone dental treatments [15]. Additionally, the general feeling of anxiety and fear had a substantial impact on the working activity of pediatric dentists [12].

It is well known that the emotional state of children is often influenced by that of their parents [16]. During pandemic situations, such as the H1N1 outbreak, it is not surprising that anxiety levels in adults increase. In this survey, it was found that anxiety in children during dental treatment has also increased. This finding contrasts with the research conducted by Olszewska et al. [17], who reported no significant difference in children’s anxiety levels compared to the pre-pandemic period. However, it is important to note that the North of Italy was severely affected by the initial wave of Coronavirus infection, which could have contributed to heightened fears of contracting an infection during a dental appointment.

Considering these findings, it should be beneficial for dental practitioners, especially in pediatric dentistry, to improve remote communication tools and to propose new methods of treatment. This may involve the use of special platforms, as well as providing practical teaching guides for parents and children [11].

The implementation of measures to reduce the spread of the virus, such as social distancing, lockdowns, and the suspension of school and sports activities, has resulted in a new routine that has significantly impacted children’s physical and psychological habits [13,18,19,20]. More than half of the pediatric dentists surveyed, particularly in the North of Italy, observed a noticeable deterioration in children’s dietary habits and oral hygiene. The psychological and emotional responses to the pandemic can increase the risk of developing unhealthy eating behaviors, such as increased consumption of high-carbohydrate snacks and junk foods [19,20,21]. A recent study conducted in Italy by Di Renzo et al. [19] reported lifestyle and dietary changes during the COVID-19 pandemic, including a shift towards convenience foods, ready-to-eat cereals, and reduced intake of fresh foods, fruits, fish and vegetables. Additionally, the altered lifestyle resulting from quarantine measures has led to decreased physical activity, increased use of electronic devices, disrupted appetite regulation, sedentary behaviors, and alterations in daily routines, including sleep patterns [19].

This survey findings also indicated a connection between sleep disorders and poor oral hygiene in children, which aligns with the results of a study by Baptista et al. in 2021 [22]. Since tooth brushing and flossing are typically integrated into daily routines, such as after breakfast and before bedtime, the disruption of these routines due to sleep disturbances can have a negative impact on oral hygiene. This creates a vicious circle where the irregular sleep–wakefulness cycle leads to a change in the fixed time for oral hygiene [23,24].

One of the limitations of this study is that a significant number of the responders were from Northern Italy, which was one of the areas most affected by the pandemic. This may have been a result of the survey distribution method through the authors’ mailing lists. Although the sample may not represent the entire population of pediatric dentists, it still provides a comprehensive overview of the changes in dental approaches to children during a pandemic. Another limitation is the response rate, which was approximately 30% of Italian pediatric dentists. It is important to consider that dentists have been receiving numerous questionnaires for other surveys since the beginning of the pandemic, which may have reduced their motivation to participate. Finally, the average age of the respondents was relatively young, with a majority under the age of 31. It would have been interesting to explore potential differences in approaches between younger and older colleagues, but this could not be verified in our study.

With the COVID-19 pandemic, dental education and the training of future pediatric dentists have also been profoundly affected. Government restrictions have led to the suspension of conventional teaching and limited clinical activities to emergency treatment only. As a result, it has been necessary to modify and adapt dental education programs [25]. This study was also used as an educational tool for dental students specializing in pediatric dentistry. The students were able to actively participate in the survey and gain valuable insights into the impact of the COVID-19 pandemic on pediatric dental care. By engaging in this learning method, the students were able to employ a problem analysis approach, from recognizing the challenges faced by pediatric dentists to proposing potential solutions. The results of the survey provided the students with data that they could analyze and discuss. They also had the opportunity to apply their knowledge and creativity to develop innovative solutions to address these challenges and propose new oral health education programs for parents and children. Additionally, the students explored the use of remote communication and monitoring tools to enhance patient care and engagement.

Distance education has proven to be as effective as traditional face-to-face education in both dental and medical fields. It offers students an autonomous and flexible learning environment, promoting creativity, critical thinking skills, and a sense of responsibility. However, there are several limitations to consider regarding this study’s applicability to dentistry students. Firstly, this survey primarily focused on pediatric dentists’ perspectives and their experiences during the COVID-19 pandemic, rather than directly assessing the educational outcomes for dentistry students. Therefore, the direct impact on dentistry students’ learning and training cannot be fully determined.

Additionally, the study does not assess the effectiveness or comparative advantages of distance education or remote learning for dentistry students specifically. More research is needed to evaluate the specific educational outcomes and challenges faced by dentistry students in remote learning environments.

## 5. Conclusions

This survey provides a comprehensive overview of the impact of the COVID-19 pandemic on pediatric dentistry in Italy, with a particular focus on heavily affected regions, such as the North. It aligns with similar studies conducted worldwide and sheds light on the various effects of the pandemic on children’s oral health and overall wellbeing. The findings reveal significant changes in pediatric dentistry during the pandemic, including a decrease in the number of pediatric treatments, a decline in oral hygiene practices, deterioration of dietary habits, and increased anxiety levels during dental procedures. These observations underscore the importance of dental practitioners and caregivers in addressing these challenges and providing comprehensive oral health education and support to children during these unprecedented times. Pediatric dentists, armed with the knowledge and experience gained during the pandemic and informed by the relevant literature, are well positioned to adapt their practice effectively to meet these new challenges.

## Figures and Tables

**Table 1:** Respondents’ characteristics.

**Respondents**	1752
Gender	
Male	274 (15.6)
Female	1478 (84.4)
Age	
Less than 30 years	692 (39.5)
31–49 years	855 (48.8)
50–60 years	178 (10.2)
Over 61 years	27 (1.5)
Dentists’ Experience	
Low (<5 years)	692 (39.5)
Middle (5–20 years)	855 (48.8)
High (≥20 years)	205 (11.7)
Working Position	
Dental associate	964 (55.0)
Dental employee	61 (3.5)
Medical director	57 (3.3)
Dental owner	670 (38.2)
Regions	
North Italy	1148 (65.5)
Other regions	604 (34.5)
Number of paediatric patients treated weekly (on average)	
Less than 6 patients	364 (20.8)
6–10 patients	430 (24.5)
More than 10 patients	958 (54.7)
Positive patients treated after the first Italian lockdown	61 (3.5)

**Table 2:** Management of dental offices during the first Italian lockdown.

	Tot. Sample	Geographic Area			Experience				Working Position				
		North Italy	Other Regions	*p*	Low	Middle	High	*p*	Dental Associate	Dental Employee	Medical Director	Dental Owner	*p*
Respondents treating dental urgencies during the first Italian lockdown													
	1196 (68.3)	756 (65.9)	440 (72.8)	0.000	478 (69.1)	545 (63.7)	173 (84.4)	0.000	720 (74.7)	30 (49.2)	30 (52.6)	416 (62.1)	0.000
Number of urgencies handled during the first Italian lockdown													
1–5	980 (81.9)	661 (87.4)	319 (72.5)	0.000	413 (86.4)	424 (77.8)	143 (82.7)	0.000	655 (91.0)	30 (100.0)	30 (100.0)	265 (63.7)	0.000
6–10	182 (15.2)	61 (8.1)	121 (27.5)		31 (6.5)	121 (22.2)	30 (17.3)		31 (4.3)	0 (0.0)	0 (0.0)	151 (36.3)	
>10	34 (2.8)	34 (4.5)	0 (0.0)		34 (7.1)	0 (0.0)	0 (0.0)		34 (4.7)	0 (0.0)	0 (0.0)	0 (0.0)	
Change in the number of requests for dental alveolar trauma management													
Increase	185 (10.6)	95 (8.3)	90 (14.9)	0.000	67 (9.7)	118 (13.8)	0 (0.0)	0.000	95 (9.9)	0 (0.0)	0 (0.0)	90 (13.4)	0.000
Decrease	485 (27.7)	390 (34.0)	95 (15.7)		157 (23.0)	241 (28.2)	87 (42.4)		337 (35.0)	0 (0.0)	27 (47.4)	121 (18.1)	
No Change	1082 (61.8)	663 (57.8)	419 (69.4)		468 (68.0)	496 (58.0)	118 (57.6)		532 (55.2)	61 (100.0)	30 (52.6)	459 (68.5)	

Legend: Total (*n* = 1752); Geographic areas: North Italy (*n* = 1148), Other regions (*n* = 604); Experience: low (*n* = 692), middle (*n* = 855), high (*n* = 205); Working position: Dental associates (*n* = 964), Dental Employees (*n* = 61), Medical Directors (*n* = 57), Dental Owners (*n* = 670).

**Table 3:** Management of paediatric patients after the lockdown.

	Tot. Sample	Geographic Area			Experience				Working Position				
		North Italy	Other Regions	*p*	Low	Middle	High	*p*	Dental Associate	Dental Employee	Medical Director	Dental Owner	*p*
Reduction of patients													
	377 (21.5)	279 (24.3)	98 (16.2)	0.000	189 (27.3)	158 (18.5)	30 (14.6)	0.000	222 (23.0)	30 (49.2)	0 (0.0)	125 (18.7)	0.000
Questions during telephone triage													
Total	1174 (67.0)	878 (76.5)	296 (49.0)	0.000	564 (81.5)	495 (57.9)	115 (56.1)	0.000	778 (80.7)	0 (0.0)	57 (100.0)	339 (50.6)	0.000
Sometimes	1050 (89.4)	757 (86.2)	293 (99.0)		534 (94.7)	431 (87.1)	85 (73.9)		718 (92.3)	0 (0.0)	57 (100.0)	275 (81.1)	
Often	124 (10.6)	121 (13.8)	3 (1.0)		30 (5.3)	64 (12.9)	80 (69.6)		60 (7.7)	0 (0.0)	0 (0.0)	64 (18.9)	
Telephone triage and COVID-19 questionnaire for													
parents and child	1372 (78.3)	899 (78.3)	473 (78.3)	0.064	464 (67.1)	790 (92.4)	118 (57.6)	0.000	737 (76.5)	30 (49.2)	57 (100.0)	548 (81.8)	0.000
only child	61 (3.5)	32 (2.8)	29 (4.8)		0 (0.0)	34 (4.0)	27 (13.2)		0 (0.0)	0 (0.0)	0 (0.0)	61 (9.1)	
only parents	319 (18.2)	217 (18.9)	102 (16.9)		228 (32.9)	31 (3.6)	60 (29.3)		227 (23.5)	31 (50.8)	0 (0.0)	61 (9.1)	
During treatment, parents remain in:													
waiting room	450 (25.7)	265 (23.1)	185 (30.6)	0.002	155 (22.4)	208 (24.3)	87 (42.4)	0.000	213 (22.1)	31 (50.8)	27 (47.4)	179 (26.7)	0.000
dental treatment rooms as usual	311 (17.8)	217 (18.9)	94 (15.6)		30 (4.3)	223 (26.1)	58 (28.3)		61 (6.3)	0 (0.0)	0 (0.0)	250 (37.3)	
dental treatment rooms only if requested	991 (56.6)	666 (58.0)	325 (53.8)		507 (73.3)	424 (49.6)	60 (29.3)		690 (71.6)	30 (49.2)	30 (52.6)	241 (36.0)	
Use of PPE during paediatric treatments:													
Yes, always	1481 (84.5)	949 (82.7)	532 (88.1)	0.003	629 (90.9)	734 (85.8)	118 (57.6)	0.000	812 (84.2)	61 (100.0)	57 (100.0)	551 (82.2)	0.000
Only for aerosol-generating procedure	271 (15.5)	199 (17.3)	72 (11.9)		63 (9.1)	121 (14.2)	87 (42.4)		152 (15.8)	0 (0.0)	0 (0.0)	119 (17.8)	
Prevalence of requests:													
First visit	333 (19.0)	179 (15.6)	154 (25.5)	0.000	124 (17.9)	209 (24.4)	0 (0.0)	0.000	182 (18.9)	0 (0.0)	27 (47.4)	124 (18.5)	0.000
Dental urgency	340 (19.4)	246 (21.4)	94 (15.6)		96 (13.9)	187 (21.9)	57 (27.8)		127 (13.2)	30 (49.2)	0 (0.0)	183 (27.3)	
Orthodontic urgency	31 (1.8)	0 (0.0)	31 (5.1)		31 (4.5)	0 (0.0)	0 (0.0)		0 (0.0)	31 (50.8)	0 (0.0)	0 (0.0)	
Orthodontic evaluation	213 (12.2)	149 (13.0)	64 (10.6)		32 (4.6)	121 (12.2)	60 (29.3)		152 (15.8)	0 (0.0)	0 (0.0)	61 (9.1)	
Control visit	835 (47.7)	574 (50.0)	261 (43.2)		409 (59.1)	338 (39.5)	88 (42.9)		503 (52.2)	0 (0.0)	30 (52.6)	302 (45.1)	

Legend: Total (*n* = 1752); Geographic areas: North Italy (*n* = 1148), Other regions (*n* = 604); Experience: low (*n* = 692), middle (*n* = 855), high (*n* = 205); Working position: Dental Associates (*n* = 964), Dental Employees (*n* = 61), Medical Directors (*n* = 57), Dental Owners (*n* = 670).

## Data Availability

The data used to support this study are available from the corresponding author upon request.

## References

[1] Tysiąc-Miśta M., Dziedzic A. (2020). The Attitudes and Professional Approaches of Dental Practitioners during the COVID-19 Outbreak in Poland: A Cross-Sectional Survey. Int. J. Environ. Res. Public Health.

[2] Bardellini E., Amadori F., Veneri F., Conti G., Majorana A. (2020). Coronavirus Disease-2019 and dental practice: A project on the use of ozonized water in the water circuit of the dental armchair. Stomatologija.

[3] Baudet A., Lizon J., Martrette J.M., Camelot F., Florentin A., Clément C. (2019). Dental Unit Waterlines: A Survey of Practices in Eastern France. Int. J. Environ. Res. Public Health.

[4] Giacomuzzi M., Zotti C.M., Ditommaso S. (2019). Colonization of Dental Unit Waterlines by *Helicobacter pylori*: Risk of Exposure in Dental Practices. Int. J. Environ. Res. Public Health.

[5] Giudice A., Bennardo F., Antonelli A., Barone S., Fortunato L. (2020). COVID-19 is a New Challenge for Dental Practitioners: Advice on Patients’ Management from Prevention of Cross Infections to Telemedicine. Open Dent. J..

[6] Cagetti M.G., Cairoli J.L., Senna A., Campus G. (2020). COVID-19 Outbreak in North Italy: An Overview on Dentistry. A Questionnaire Survey. Int. J. Environ. Res. Public Health.

[7] Khader Y., Al Nsour M., Al-Batayneh O.B., Saadeh R., Bashier H., Alfaqih M., Al-Azzam S., AlShurman B.A. (2020). Dentists’ Awareness, Perception, and Attitude Regarding COVID-19 and Infection Control: Cross-Sectional Study Among Jordanian Dentists. JMIR Public Health Surveill.

[8] Bontà G., Campus G., Cagetti M.G. (2020). COVID-19 pandemic and dental hygienists in Italy: A questionnaire survey. BMC Health Serv. Res..

[9] Cimilluca J.J., Lee K.C., Halepas S., Ferguson B. (2021). COVID-19 Pandemic and its Impact on Dentistry: A Cross-sectional Survey of Practicing Dentists. J. Contemp. Dent. Pract..

[10] COVIDental Collaboration Group (2021). The COVID-19 pandemic and its global effects on dental practice. An International survey. J. Dent..

[11] Luzzi V., Ierardo G., Bossù M., Polimeni A. (2021). Paediatric Oral Health during and after the COVID-19 Pandemic. Int. J. Paediatr. Dent..

[12] Hopcraft M., Farmer G. (2021). Impact of COVID-19 on the provision of paediatric dental care: Analysis of the Australian Child Dental Benefits Schedule. Community Dent. Oral Epidemiol..

[13] Cotrin P., Peloso R.M., Oliveira R.C., de Oliveira R.C.G., Pini N.I.P., Valarelli F.P., Freitas K.M.S. (2020). Impact of coronavirus pandemic in appointments and anxiety/concerns of patients regarding orthodontic treatment. Orthod. Craniofac. Res..

[14] Campagnaro R., Collet G.O., Andrade M.P., Salles J.P.D.S.L., Calvo Fracasso M.L., Scheffel D.L.S., Freitas K.M.S., Santin G.C. (2020). COVID-19 pandemic and paediatric dentistry: Fear, eating habits and parent’s oral health perceptions. Child Youth Serv. Rev..

[15] Peloso R.M., Pini N.I.P., Sundfeld Neto D., Mori A.A., Oliveira R.C.G., Valarelli F.P., Freitas K.M.S. (2020). How does the quarantine resulting from COVID-19 impact dental appointments and patient anxiety levels?. Braz. Oral Res..

[16] Dopp A.R., Cain A.C. (2012). The role of peer relationships in parental bereavement during childhood and adolescence. Death Stud..

[17] Olszewska A., Rzymski P. (2020). Children’s Dental Anxiety during the COVID-19 Pandemic: Polish Experience. J. Clin. Med..

[18] Creswell C., Nauta M.H., Hudson J.L., March S., Reardon T., Arendt K., Bodden D., Cobham V.E., Donovan C., Halldorsson B. (2021). Research Review: Recommendations for reporting on treatment trials for child and adolescent anxiety disorders—An international consensus statement. J. Child Psychol. Psychiatry.

[19] Di Renzo L., Gualtieri P., Pivari F., Soldati L., Attinà A., Cinelli G., Leggeri C., Caparello G., Barrea L., Scerbo F. (2020). Eating habits and lifestyle changes during COVID-19 lockdown: An Italian survey. J. Transl. Med..

[20] Spinelli M., Lionetti F., Pastore M., Fasolo M. (2020). Parents’ Stress and Children’s Psychological Problems in Families Facing the COVID-19 Outbreak in Italy. Front. Psychol..

[21] Wang C., Pan R., Wan X., Tan Y., Xu L., Ho C.S., Ho R.C. (2020). Immediate Psychological Responses and Associated Factors during the Initial Stage of the 2019 Coronavirus Disease (COVID-19) Epidemic among the General Population in China. Int. J. Environ. Res. Public Health.

[22] Baptista A.S., Prado I.M., Perazzo M.F., Pinho T., Paiva S.M., Pordeus I.A., Serra-Negra J.M. (2021). Can children’s oral hygiene and sleep routines be compromised during the COVID-19 pandemic?. Int. J. Paediatr. Dent..

[23] Trubey R.J., Moore S.C., Chestnutt I.G. (2014). Parents’ reasons for brushing or not brushing their child’s teeth: A qualitative study. Int. J. Paediatr. Dent..

[24] Bardellini E., Bondioni M.P., Amadori F., Veneri F., Lougaris V., Meini A., Plebani A., Majorana A. (2021). Non-specific oral and cutaneous manifestations of Coronavirus Disease 2019 in children. Med. Oral Patol. Oral Cir. Bucal..

[25] Abbasi M.S., Ahmed N., Sajjad B., Alshahrani A., Saeed S., Sarfaraz S., Alhamdan R.S., Vohra F., Abduljabbar T. (2020). E-Learning perception and satisfaction among health sciences students amid the COVID-19 pandemic. Work.

